# Characterization of the abomasal transcriptome for mechanisms of resistance to gastrointestinal nematodes in cattle

**DOI:** 10.1186/1297-9716-42-114

**Published:** 2011-11-30

**Authors:** Robert W Li, Manuela Rinaldi, Anthony V Capuco

**Affiliations:** 1 Bovine Functional Genomics Laboratory, Animal and Natural Resources Institute, USDA-ARS, Beltsville, MD 20705, USA; 2 Department of Virology, Parasitology and Immunology, Faculty of Veterinary Medicine, Ghent University, Belgium

## Abstract

The response of the abomasal transcriptome to gastrointestinal parasites was evaluated in parasite-susceptible and parasite-resistant Angus cattle using RNA-seq at a depth of 23.7 million sequences per sample. These cattle displayed distinctly separate resistance phenotypes as assessed by fecal egg counts. Approximately 65.3% of the 23 632 bovine genes were expressed in the fundic abomasum. Of these, 13 758 genes were expressed in all samples tested and likely represent core components of the bovine abomasal transcriptome. The gene (BT14427) with the most abundant transcript, accounting for 10.4% of sequences in the transcriptome, is located on chromosome 29 and has unknown functions. Additionally, PIGR (1.6%), Complement C3 (0.7%), and Immunoglobulin J chain (0.5%) were among the most abundant transcripts in the transcriptome. Among the 203 genes impacted, 64 were significantly over-expressed in resistant animals at a stringent cutoff (FDR < 5%). Among the 94 224 splice junctions identified, 133 were uniquely present: 90 were observed only in resistant animals, and 43 were present only in susceptible animals. Gene Ontology (GO) enrichment of the genes under study uncovered an association with lipid metabolism, which was confirmed by an independent pathway analysis. Several pathways, such as FXR/RXR activation, LXR/RXR activation, LPS/IL-1 mediated inhibition of RXR function, and arachidonic acid metabolism, were impacted in resistant animals, which are potentially involved in the development of parasite resistance in cattle. Our results provide insights into the development of host immunity to gastrointestinal nematode infection and will facilitate understanding of mechanism underlying host resistance.

## Introduction

Gastrointestinal (GI) nematode infections in ruminants remain a major impediment to the efficient production of both meat and dairy products, and therefore, represent a major constraint on global food availability. These GI infections have a significant economic impact on the U.S. cattle industry, with an estimated annual cost of ~$2 billion per year in lost productivity and increased operating expenses. Nematode infections of the GI tract impact numerous production traits. Among the most prominent effect is a reduction in weight gain that may cause decreased bodyweights of up to 14% [1]. Although the impact is particularly evident in young calves, substantial evidence suggests that infection produces long lasting effects on the productivity of adult cattle [2]. In dairy cows, parasitic infections reduce milk yield between 1.2 and 2.2 kg milk/cow per day [3]. Infections also negatively impact carcass quality and reproductive performance, including calving rate and calf mortality [4]. Potential economic loss resulting from GI nematode infections is clearly recognized by producers and veterinarians, as evidenced by the fact that approximately 99% of feedlots and 69% of dairies use a parasiticide in their operations [5].

Among 41 bovine GI nematodes, species from the genera *Ostertagia*, *Cooperia*, and *Nematodirus *are arguably the most important cattle parasites in temperate regions of the world, as assessed by their negative economic impact [6]. Development of protective immunity and resistance to these GI nematodes relies upon the precise control of expression of the host genome. It is evident that the evolution of regulatory programs controlling the transcriptome occurs at a rapid rate comparable to that of other genomic processes. Understanding these regulatory elements is crucial towards unraveling their functional relevance. Comparative transcriptomic analysis has emerged as a promising means for unraveling the molecular basis and regulatory networks underlying complex traits such as host resistance. While recent progress has been made with regard to genes associated with nematode resistance in small ruminants [7-9], an in-depth comparison and characterization of transcriptomic responses of cattle populations that harbor varying degrees of resistance to parasitic nematodes is not yet available. Sequencing steady-state RNA in a biological sample (RNA-seq technology) using next-generation sequencing platforms (e.g. Illumina) overcomes many limitations of previous technologies, such as microarrays and real-time PCR. Most importantly, RNA-seq has been shown to elucidate previously inaccessible complexities in the transcriptome, such as allele-specific expression and involvement of novel promoters and isoforms [10], detection of alternative splicing [11], RNA editing [12], novel transcripts [13], all in conjunction with quantitative evaluation of transcript abundance [14]. In this study, we utilized RNA-seq technology to characterize the bovine transcriptome response of nematode-resistant and nematode-susceptible heifers to identify molecular mechanisms that underlie host resistance to GI nematodes in cattle.

## Materials and methods

### Animals and parasitology

Six 12-month-old Angus heifers that differed with regard to susceptibility to GI nematode infection (3 resistant and 3 susceptible) were used in this experiment. These heifers were from a selective breeding program for parasite resistance that was initiated at our facilities in 1991, using parental stock originating from the Wye Angus herd [15,16]. Once the initial breeding females were identified, semen from high and low EPG (eggs per gram of feces) bulls was used to produce calves of desired phenotypes. Calves were kept with their dams on pastures with extremely low numbers of parasites prior to weaning. When the median age of the contemporary group was 205 days, calves were weaned and placed on pastures infected with the two most common nematode parasites of cattle, *Ostertagia ostertagi *and *Cooperia oncophora*. The calves were monitored weekly for a number of parasitologic and immunologic parameters along with selected measurements of animal growth. The calves were kept pastured for a minimum of 120 days. Replacement animals were selected for secondary challenge experiments, while parasitologic and immunologic parameters were collected from animals chosen for slaughter. This program resulted in resource populations selected for the fecal egg trait (high or low fecal eggs counts or eggs per gram, EPG; high or low parasite resistance, respectively). Based on actual weekly EPG counts and sire expected progeny difference (EPD) values for EPG, a total of six heifers were selected for this study. Three heifers used in this study were classified as susceptible (high EPG EPD value) and the remaining 3 were classified as resistant (low EPG EPD value).

At the end of the grazing season, all heifers were treated with a combination of 10 mg fenbendazole and 0.5 mg moxidectin per kg of body weight to remove existing GI parasites transmitted from the infected pastures. After resting for 30 days on concrete to preclude further parasite exposure, the heifers were orally infected with a single dose of combined *O. ostertagi *and *C. oncophora *infective L3 larvae (8.5 × 10^4 ^and 1.0 × 10^5 ^*O. ostertagi *and *C. oncophora *L3 larvae, respectively, per animal) and housed on concrete for an additional 20 days, allowing the experimental infection to progress. EPG was monitored during the resting period to ensure that the drug treatment eliminated all pre-existing parasites. The infective L3 larvae were obtained from cultures maintained at the USDA-ARS Beltsville facilities. The heifers were handled according to a protocol approved by the Beltsville Agricultural Research Center Animal Care and Use Committee, following Institutional Animal Care and Use Committees (IACUC) guidelines. The heifers were sacrificed at 20 days post infection (dpi). Worms were counted from the contents of the abomasum and small intestine. The full-thickness of folds from the fundic abomasa were collected, minced into 1-2 cm pieces, and snap frozen in liquid nitrogen prior to storage at -80°C until total RNA was extracted.

### RNA extraction and sequencing using RNA-seq technology

Total RNA was extracted using Trizol followed by DNase digestion and Qiagen RNeasy column purification as previously described [17]. The RNA integrity was verified using an Agilent Bioanalyzer 2100 (Agilent, Palo Alto, CA, USA) with a RIN value > 7.0. High-quality RNA was processed using an Illumina RNA-seq sample prep kit following the manufacturer's instruction (Illumina, San Diego, CA, USA). Final RNA-seq libraries were validated and sequenced at 36bp/sequence read using an Illumina GAIIx sequencer at a depth of approximately 23.7 million sequences per sample (mean ± SD = 23 723 620 ± 7 447 499 per sample).

### Data analysis and bioinformatics

23 632 bovine genes in the Bovine Official Gene Set version 2 (OGS2.0) [18] were first mapped to the bovine reference genome (Btau4.0) using Genomic Short-read Nucleotide Alignment Program or GSNAP [19]. The best mapping position of each gene (≤ 10 kb intron span, ≥ 95% identity, ≥ 90% coverage, and minimum tail length of 5% of coding sequences) was extracted. Accordingly, 20 809 of the 23 632 bovine genes were uniquely mapped. After removing ambiguously mapped genes, 18 834 genes were used for RNA-seq data analysis.

Raw sequence reads were then checked using several layers of quality control filtering to remove low-quality reads. Raw reads with ≥ 2 ambiguous nucleotides (N) were discarded. Trimming removed approximately 1% of input raw reads and led to the retention of 99% of raw reads. The input reads after cleansing were mapped to the reference genome with gene coordinates using Bowtie (v0.12.7), an ultrafast and memory-efficient short-read aligner using a Burrows-Wheeler index [20]. Approximately 68.5% of trimmed reads mapped to the bovine genome (mean ± SD = 68.54% ± 2.48%). Only reads with one unique best match in the reference genome were used for subsequent analyses. The read depth of each gene was computed based on the coordinates of mapped reads and gene locations in the reference genome and was normalized using a method that corrects for biases introduced by RNA composition and differences in the total numbers of uniquely mapped reads in each sample [21]. Only genes having ≥ 20 uniquely mapped reads (mean of all 6 samples) were further analyzed. The R package *edgeR *was used to test the null hypothesis that expression of a given gene is not different between the two groups [21]. The normalized read counts were also analyzed using the DEGseq algorithm [22]. The DEGseq built-in function "samWrapper" that is recommended for testing RNA-seq data with biological replication was used to detect differential expression. Candidate genes were first sorted based on *P *value (*P *< 0.05) and fold change (2-fold as a cutoff). Genes identified as candidates for differential expression were further filtered with a false discovery rate (FDR) of < 5% to account for multiple testing. Differentially-expressed genes in the transcriptome were further analyzed using Gene Ontology (GO) analysis (GOseq). Over-representation of certain GO terms was determined based on Fisher's exact test. A multiple correction control (permutation to control false discovery rate [23]) was implemented to set up the threshold to obtain the lists of significantly over-represented GO terms. The candidate genes were analyzed using IPA v9.0 for pathways (Ingenuity Systems, Redwood City, CA, USA).

Tophat (v1.2.0) was used to map input reads to the reference genome [11] and identify potential splice junctions or splicing variants. Only reads with one unique match in the reference genome were used for subsequent analyses. The maximum allowed intron size was 5kb (a conservative parameter to avoid a high false discovery rate). At each potential splice junction, spanning reads were counted. Potential splice junctions were compared to annotated splice sites. To identify differential splice junctions between two groups, normalized read counts of splice junctions were required to be eight times different between resistant and susceptible groups. An unpaired *t*-test was performed on normalized sequence read counts. Splice junctions at *P *≤ 0.05 were considered candidates junctions that were differentially regulated between resistant and susceptible groups.

### Real-time RT-PCR

Real-time or quantitative RT-PCR (qPCR) was performed as previously described [17]. Briefly, the cDNA synthesis was performed using an iScript cDNA Synthesis kit (Bio-Rad, Hercules, CA, USA). Real-time RT-PCR analysis was carried out with an iQ SYBR Green Supermix kit (Biorad) using 200 nM of each amplification primer and the 1^st^-strand cDNA (100 ng of the input total RNA equivalents) in a 25 μL reaction volume as described. The amplification was carried out on an iCycler iQ™ Real Time PCR Detection System (BioRad) with the following profile: 95°C-60 s; 40 cycles of 94°C-15 s, 60°C-30 s, and 72°C-30 s. A melting curve analysis was performed for each primer pair. The gene encoding for phospholipase A2, group IVA (cytosolic, calcium-dependent) (PLA2G4A), which has a relatively constant expression level across all experiment samples, was used as an endogenous control. Relative gene expression data was calculated using the 2^-ΔΔCT ^method. The fold change was normalized against the susceptible group.

## Results

### EPG and worm counts

**Figure 1:**
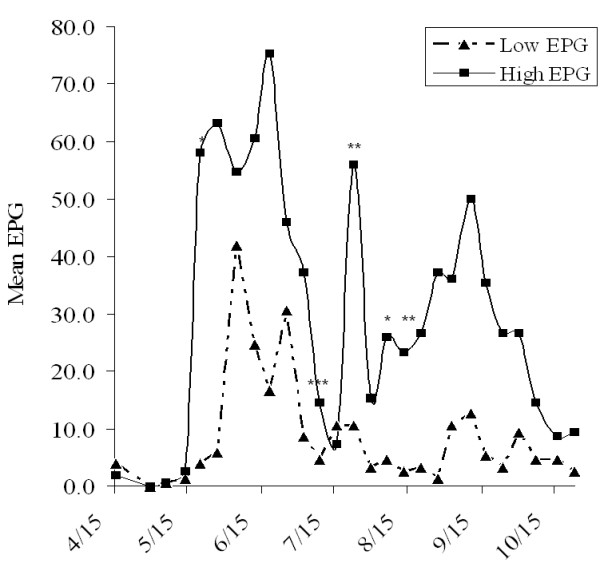
**Weekly mean fecal egg counts (eggs per gram, EPG or FEC) of resistant and susceptible Angus heifers grazing on infected pasture between April to October**. Y-axis represents mean weekly fecal egg counts. * indicates a significant difference in EPG between resistant and susceptible groups (*P *< 0.05). ** *P *< 0.01; *** *P *< 0.001.

Mean weekly EPG values were 8.8 ± 1.6 (Mean ± SD) and 31.3 ± 10.4 for low-EPG (resistant) and high-EPG (susceptible) heifers, respectively (*N *= 3) during the 6-month grazing season (Figure 1). In accordance with the experimental design, this difference was statistically significant (*P *< 0.05). Although a temporal fluctuation in weekly EPG values was evident, resistant heifers shed constantly fewer parasite eggs in feces than did susceptible heifers. Over the grazing period, resistant heifers gained more weight (*P *< 0.05) than susceptible heifers (159.0 ± 32.8 vs 92.1 ± 20.4 lbs). Similarly, resistant heifers displayed a numerically, but not statistically, greater (*P *> 0.05) gain in hip height over the experiment period (Table 1). Serum pepsinogen levels between both resistant and susceptible groups were statistically indistinguishable. The mean number of total parasite worms (both *O. ostertagi *and *C. oncophora*) recovered from resistant heifers (5 200 ± 3 191) after the experimental challenge were numerically less than those of susceptible heifers (5 923 ± 3 203), but not significantly less (*P *> 0.05). These worm burden data were not unexpected because the population under study has never been selected for worm burdens as an indicator trait in the applied breeding program. Additionally, the worm counts were obtained from an experimental infection with a high dose of infective larvae that is not typically encountered by calves under nature exposure.

**Table 1:** Growth and parasitology parameters between parasite-resistant or susceptible cattle.

	Resistant	Susceptible
	(Low-EPG)	(High-EPG)
During grazing period:		
EPG (weekly mean)	8.8 ± 1.6*	31.3 ± 10.4
Weight gain (lb)	159.0 ± 32.8*	92.1 ± 20.4
Hip height gain (cM)	8.26 ± 1.10	6.35 ± 2.54
Pepsinogen (mU)	689.0 ± 20.0	672.5 ± 230.0
Post experimental infection:		
Worm count	5200 ± 3191	5923 ± 3203
		
Mean ± SD (*N *= 3)		
*P < 0.05		

### General characteristics of the bovine abomasal transcriptome

20 809 of the 23 633 bovine genes (88%) were uniquely mapped to the bovine genome (Bta4.0). Among these, 18 834 genes were unambiguously mapped and were used for RNA-seq analysis. 14 549 to 15 432 of the 18 834 genes had at least one copy of their transcripts expressed in the bovine abomasal transcriptome (61.6 to 65.3% of all bovine genes). 11 474 to 13 015 genes had ≥ 10 sequence hits in the bovine abomasal tissue. 13 758 genes were expressed in all bovine abomasal samples tested, probably representing the core component of the bovine abomasal transcriptome. The most abundant transcript in all 6 abomasal samples tested was a gene (Gene ID: BT14427) located on *Bos taurus *autosome (BTA or chromosome) 29 whose function is unknown but represents 10.38% of sequence reads in the transcriptome. The next most abundant transcripts included an unknown gene (BT10810, 2.36%), polymeric immunoglobulin receptor (PIGR, 1.60%), a gene on BTA11 (BT28533, 1.29%), complement C3 (0.73%), growth arrest-specific protein 7 (0.67%), pre-B lymphocyte protein 3 (0.60%), liver fatty acid-binding protein (0.56%), IgG Fc-binding protein (0.53%), and immunoglobulin J chain (0.49%). The 10 most abundant protein-coding genes accounted for 19.21% of all sequence reads in the bovine abomasal transcriptome.

### Differentially expressed genes

Normalized sequence counts were analyzed using both *edgeR *and DEGseq algorithms. A total of 203 genes met 2 criteria: unadjusted *P *value < 0.05 and 2-fold difference in normalized read counts between resistant and susceptible animals (Additional file 1). These candidate genes were further filtered with a stringent cutoff (FDR < 5%). Sixty four genes were significantly different between resistant and susceptible animals at FDR < 5% (Table 2). These genes had a significantly higher ratio of normalized sequence counts between resistant and susceptible groups. For example, common salivary protein BSP10, form A (BT12506 or SPLUNC2A) was expressed 57.68 fold higher in resistant heifers. Similarly, the sequence counts of intelectin (ITLN2) in the abomasum of resistant heifers were 51.98 times higher than in that of susceptible heifers. Mucin 12 (MUC12) and fatty acid binding protein 6, ileal (FABP6, intestinal bile acid-binding protein or gastrotropin) were significantly over-expressed in resistant animals. Several apolipoproteins (APOA1, A4, B100, and C2) were also over-expressed in resistant heifers. Transcripts for alpha-inducible protein 27 and 27-like 2 (IFI27 and IFI27L2) were more abundant in resistant than in susceptible animals.

**Table 2:** Genes significantly regulated during parasitic infections in resistant cattle.

ID Symbol	BTA	Start	End	Ratio	P value FDR	Resistant RPKM	Susceptible RPKM
BT23148 ACCN3	Chr4	117903455	117907312	12.73	0.000 0.0000	3.97 ± 6.61	0.27 ± 0.06
BT15243 ANPEP	Chr21	20951416	20967921	11.08	0.000 0.0000	40.93 ± 28.64	3.67 ± 0.80
BT22521 APOA1	Chr15	25933779	25935362	5.98	0.000 0.0000	5360.53 ± 1986.27	895.53 ± 533.24
BT12506 SPLUNC2A	Chr13	63394161	63402279	57.68	0.000 0.0000	4.80 ± 6.20	0.10 ± 0.10
BT25183 CSMD2	Chr3	118882335	119566628	9.58	0.000 0.0000	1.23 ± 0.65	0.13 ± 0.06
BT22188 CYP4B1	Chr3	106240610	106260595	7.21	0.000 0.0000	4.67 ± 1.85	0.63 ± 0.15
BT26217 DUOX2	Chr10	66934451	66952107	12.47	0.000 0.0000	10.27 ± 14.53	0.87 ± 0.55
BT27848 FABP6	Chr7	71616618	71622552	8.34	0.000 0.0000	20.10 ± 29.36	2.23 ± 0.45
BT22964 ITLN2	Chr3	9558554	9565419	51.98	0.000 0.0000	18.37 ± 29.41	0.37 ± 0.47
BT19368	Chr25	4742693	4775217	6.96	0.000 0.0000	82.73 ± 63.48	11.77 ± 8.64
BT10244 PRSS2	Chr4	110025504	110029213	57.68	0.000 0.0000	17.93 ± 30.63	0.37 ± 0.23
BT28349 SLC6A18	Chr20	75154396	75168950	8.28	0.000 0.0000	18.87 ± 13.67	2.27 ± 0.70
BT11110	Chr13	59373661	59378915	5.86	0.000 0.0000	36.77 ± 10.25	6.30 ± 1.87
BT18264	Chr2	124281340	124288387	8.94	0.000 0.0000	2.87 ± 2.00	0.33 ± 0.12
BT26677 MRP4	ChrUn	841	156276	8.00	0.000 0.0000	12.37 ± 11.34	1.50 ± 0.78
BT29561	Chr10	66930244	66933576	22.63	0.000 0.0000	17.40 ± 27.38	0.83 ± 0.49
BT16567 APOA4	Chr15	25908269	25910771	4.44	0.000 0.0000	2075.70 ± 1258.45	466.47 ± 179.94
BT20816 APOC3	Chr15	25916846	25918590	4.59	0.000 0.0000	1467.53 ± 591.47	319.20 ± 105.66
BT20772	Chr6	87416154	87458606	5.54	0.000 0.0000	6.30 ± 5.07	1.13 ± 0.32
BT16522 MS4A10	Chr29	38877233	38884049	4.79	0.000 0.0000	75.00 ± 16.71	15.73 ± 4.97
BT13412 APOB	Chr11	80208992	80221450	4.41	0.000 0.0000	166.77 ± 21.11	37.80 ± 13.65
BT25015 ISG15	Chr16	48718864	48719323	4.47	0.000 0.0000	61.67 ± 35.27	13.63 ± 6.02
BT13211	Chr2	124265402	124268318	4.14	0.000 0.0001	149.53 ± 65.38	36.03 ± 13.41
BT26247 MAPK11	ChrUn	21657	24311	4.11	0.000 0.0001	37.87 ± 24.23	9.03 ± 4.17
BT16178	Chr23	17665934	17668511	5.06	0.000 0.0001	6.90 ± 3.24	1.30 ± 0.36
BT10335 CD36	ChrUn	11614	40612	3.97	0.000 0.0001	78.23 ± 52.91	19.73 ± 4.58
BT29523	Chr28	1263980	1431674	4.56	0.000 0.0002	3.07 ± 0.38	0.67 ± 0.64
BT30176 AKR1C3	Chr13	43830404	43853848	4.23	0.000 0.0002	14.237 ± .61	3.40 ± 0.26
BT16664	ChrX	70843101	71124091	5.28	0.000 0.0003	4.87 ± 5.93	1.03 ± 0.86
BT21051	Chr26	16748567	16803178	3.76	0.000 0.0004	26.30 ± 9.04	6.97 ± 1.55
BT28260	Chr16	48526316	48530064	4.26	0.000 0.0006	7.43 ± 2.64	1.67 ± 0.72
BT10100 KLRJ1	Chr5	106747081	106756866	3.68	0.000 0.0009	16.73 ± 6.80	4.37 ± 2.66
BT23076 HABP2	Chr26	34481504	34515729	3.84	0.000 0.0009	6.30 ± 4.06	1.57 ± 0.71
BT14255 RHOD	Chr29	46952791	46962446	3.92	0.000 0.0009	18.20 ± 13.04	4.67 ± 1.69
BT27136 MUC12	Chr25	37794638	37851653	3.41	0.000 0.0014	651.97 ± 214.37	191.10 ± 21.35
BT25367 TMEM151A	Chr29	46232359	46233687	4.11	0.000 0.0015	3.80 ± 3.18	0.83 ± 0.40
BT22967 LAMB3	Chr16	71833965	71874951	3.39	0.000 0.0016	18.07 ± 7.98	5.30 ± 1.35
BT18107	ChrUn	25167	25761	3.39	0.000 0.0016	54.57 ± 39.67	15.50 ± 23.47
BT22962 PMP22	Chr19	33646745	33669580	3.29	0.000 0.0031	70.67 ± 30.81	21.30 ± 3.36
BT12589	Chr26	9064853	9107531	3.23	0.000 0.0042	21.07 ± 11.38	6.50 ± 3.35
BT20448 STYK1	Chr5	106284244	106301611	3.41	0.000 0.0052	7.07 ± 2.12	1.97 ± 0.83
BT27305 CR2	ChrUn	83330	107409	3.66	0.000 0.0061	3.00 ± 2.87	0.77 ± 0.15
BT23649 UBD	Chr23	29024538	29026285	3.14	0.000 0.0063	48.37 ± 31.81	14.97 ± 7.41
BT21042 CLCA4	Chr3	61006123	61036756	3.03	0.000 0.0067	89.97 ± 36.33	29.57 ± 21.61
BT16585 GDPD2	ChrX	49832662	49839846	3.07	0.000 0.0071	32.20 ± 3.57	10.43 ± 1.76
BT18095 IFI27	Chr21	59045145	59052921	2.99	0.000 0.0092	109.67 ± 95.80	36.37 ± 13.02
BT23509 RSAD2	Chr11	92861557	92877381	3.20	0.000 0.0104	10.27 ± 3.46	3.17 ± 0.99
BT17415 TMEM37	Chr2	74700175	74705806	4.20	0.000 0.0107	5.53 ± 3.86	1.30 ± 0.66
BT22660 SLC7A8	Chr10	21975064	22025425	3.20	0.000 0.0119	6.20 ± 1.61	1.93 ± 0.35
BT29929	Chr10	35876816	35888464	2.91	0.000 0.0128	214.37 ± 116.87	73.60 ± 3.59
BT23760 IFI27L2	Chr21	59033630	59035497	2.95	0.000 0.0136	161.83 ± 67.72	55.17 ± 21.09
BT29480 APOC2	Chr18	52441529	52442339	3.66	0.000 0.0152	15.27 ± 8.78	4.43 ± 1.44
BT14554	Chr19	49340980	49341417	2.89	0.000 0.0263	38.03 ± 26.47	12.97 ± 4.14
BT26136 RCAN1	Chr1	327957	337475	3.05	0.000 0.0265	16.73 ± 4.79	5.60 ± 2.16
BT30154 FLVCR2	Chr10	89235896	89375608	2.87	0.000 0.0309	7.97 ± 1.66	2.73 ± 0.68
BT14279 ACE	Chr19	49341698	49380229	2.71	0.000 0.0315	55.47 ± 25.96	20.50 ± 4.80
BT10643 TRPV6	Chr4	110136393	110152623	3.18	0.000 0.0330	3.73 ± 1.35	1.30 ± 0.95
BT22748 SAMD9	Chr4	10706910	10711652	2.75	0.000 0.0396	5.33 ± 3.53	1.93 ± 0.15
BT24262 BoLA	Chr23	27786581	27806088	2.69	0.000 0.0407	59.40 ± 56.51	21.70 ± 7.07
BT12279 CYP3A4	ChrUn	25739	84849	2.71	0.000 0.0407	13.80 ± 5.60	5.00 ± 2.23
BT14013	Chr2	124213765	124262789	3.25	0.000 0.0407	1.83 ± 1.33	0.57 ± 0.23
BT13301 SECTM1	Chr19	51927819	51929433	2.73	0.000 0.0408	34.83 ± 22.23	12.57 ± 6.56
BT20562 UNC13C	Chr10	56622366	57047991	2.93	0.000 0.0461	2.47 ± 3.58	0.90 ± 0.20
BT17441 GGT1	Chr17	74684303	74693673	2.62	0.000 0.0473	35.97 ± 21.37	13.60 ± 5.19

64 of 203 differentially expressed genes at a false discovery rate FDR <5% are listed.

The numbers denote mean ± SD (N = 3). Ratio = normalized read counts of resistant animals divided by normalized read counts of susceptible animals. RPKM = reads per kilobase of exon model per million mapped reads. BTA = Bos taurus autosome (chromosome).

*Read count ratio = normalized read counts of resistant animals (Low-EPG) divided by normalized read counts of susceptible animals (High-EPG). **mean ± SD. RPKM = reads per kilobase of exon model per million mapped reads [14].

BTA = *Bos taurus *autosome (chromosome).

### Splicing variants

A total of 94 224 potential splice junctions spanned by ≥ 1 sequence read were identified using TopHat. Among them, 139 junctions displayed a significantly different number of sequence reads between resistant and susceptible groups (FDR < 5%). These 139 junctions were distributed on 28 autosomes and the X chromosome. There were no junctions on BTA12 that had significantly different numbers of sequence reads between the two groups. However, the distribution of these 139 junctions did not appear to be random, and the number of these junctions was not proportional to the physical length of the chromosomes. The vast majority of these junctions were unique to one of the two groups. For example, 90 of the 139 junctions were observed in all 3 resistant heifers but absent in 3 susceptible heifers. On the other hand, 43 were only present in susceptible heifers. Some of these unique junctions were observed in 9 of the 64 differentially expressed genes. For instance, 8 sequences (normalized mean counts) spanning an intron-exon junction (Intron position start 33 668 350 and end 33 669 502) on BTA19 were observed only in susceptible heifers and occurred inside a gene named peripheral myelin protein 2 (BT22962). A unique junction in the gene GDPD2 (BT16585) was observed only in resistant animals. The 10 most abundant unique junctions from each group are listed in Table 3.

**Table 3:** Select unique splice junctions.

GeneID	Symbol	BTA	Intron start	Intron end	Strand	P	Resistant RPKM	Susceptible RPKM
BT19086	RPLP0	17	65831745	65832562	-	0.00	191.67 ± 52.35	0.00 ± 0.00
		22	57847411	57847968	+	0.02	95.67 ± 40.62	0.00 ± 0.00
BT30349		25	15202695	15206251	-	0.01	65.33 ± 25.11	0.00 ± 0.00
BT16030		18	45802227	45803786	+	0.00	61.00 ± 14.00	0.00 ± 0.00
BT30349		25	15211963	15213263	-	0.02	41.67 ± 18.01	0.00 ± 0.00
BT16075	PDIA4	4	1.17E+08	1.17E+08	-	0.00	34.67 ± 2.89	0.00 ± 0.00
BT11599	CDH1	18	35125197	35126364	+	0.00	28.33 ± 8.08	0.00 ± 0.00
BT21452	NDUFS8	29	47590889	47590971	-	0.03	28.00 ± 14.53	0.00 ± 0.00
BT10815		18	56111950	56112091	+	0.02	25.33 ± 11.72	0.00 ± 0.00
BT16221	CAPRIN1	15	64405872	64406267	+	0.00	25.33 ± 3.21	0.00 ± 0.00
BT18538	MGST3	3	3966963	3968087	-	0.00	0.00 ± 0.00	152.00 ± 22.54
BT11164	ATP1A1	3	29338231	29338761	-	0.02	0.00 ± 0.00	143.00 ± 65.80
BT17219		7	18630456	18631070	+	0.00	0.00 ± 0.00	78.67 ± 11.50
BT24099		7	9449807	9451903	+	0.00	0.00 ± 0.00	62.67 ± 15.53
BT10200	TPI1	5	10565021	10565403	-	0.00	0.00 ± 0.00	55.67 ± 15.89
BT23745	ATP5O	1	725492	726173	+	0.01	0.00 ± 0.00	46.67 ± 15.63
BT10027	STOM	8	1.16E+08	1.16E+08	-	0.00	0.00 ± 0.00	46.33 ± 6.43
BT23715	IDH2	un004	22811	25159	+	0.00	0.00 ± 0.00	43.67 ± 9.07
BT13211		2	1.24E+08	1.24E+08	+	0.01	0.00 ± 0.00	41.00 ± 17.09
BT16976		5	1.17E+08	1.17E+08	-	0.00	0.00 ± 0.00	40.67 ± 10.50

BTA = *Bos taurus *autosome (chromosome). RPKM = reads per kilobase of exon model per million mapped reads (mean ± SD).

### Gene Ontology (GO) and pathway analyses

Over-representation of GO terms was determined based on Fisher's exact test and filtered further using a multiple correction control at FDR < 5%. As Table 4 shows, the GO enrichment of genes under study was predominantly associated with lipid metabolism.

**Table 4:** Gene Ontology (GO) associated with 64 genes that are differentially expressed.

GO ID	GO Description	Gene#	P value	FDR
GO:0042632	cholesterol homeostasis	4	1.58E-05	0.01
GO:0030301	cholesterol transport	5	3.93E-07	0.00
GO:0017127	cholesterol transporter activity	3	1.32E-05	0.01
GO:0042627	chylomicron	3	5.56E-06	0.00
GO:0005615	extracellular space	9	3.23E-05	0.05
GO:0034364	high-density lipoprotein particle	3	8.85E-06	0.01
GO:0006869	lipid transport	6	1.04E-05	0.01
GO:0042157	lipoprotein metabolic process	4	8.31E-06	0.01
GO:0042953	lipoprotein transport	3	3.19E-06	0.00
GO:0034367	macromolecular complex remodeling	4	1.11E-06	0.00
GO:0044243	multicellular organismal catabolic process	3	3.42E-05	0.05
GO:0071702	organic substance transport	9	5.81E-06	0.01
GO:0033700	phospholipid efflux	3	3.19E-06	0.00
GO:0034358	plasma lipoprotein particle	4	1.11E-06	0.00
GO:0034377	plasma lipoprotein particle assembly	3	8.85E-06	0.01
GO:0034381	plasma lipoprotein particle clearance	4	2.73E-07	0.00
GO:0071827	plasma lipoprotein particle organization	4	2.45E-06	0.00
GO:0034369	plasma lipoprotein particle remodeling	4	1.11E-06	0.00
GO:0005886	plasma membrane	20	3.44E-05	0.05
GO:0032994	protein-lipid complex	4	1.11E-06	0.00
GO:0065005	protein-lipid complex assembly	3	8.85E-06	0.01
GO:0034368	protein-lipid complex remodeling	4	1.11E-06	0.00
GO:0071825	protein-lipid complex subunit organization	4	2.45E-06	0.00
GO:0032374	regulation of cholesterol transport	3	4.43E-05	0.06
GO:0032371	regulation of sterol transport	3	4.43E-05	0.06
GO:0010901	regulation of VLDL particle remodeling	2	3.01E-05	0.02
GO:0043691	reverse cholesterol transport	3	4.43E-05	0.06
GO:0055092	sterol homeostasis	4	1.58E-05	0.01
GO:0015918	sterol transport	5	3.93E-07	0.00
GO:0015248	sterol transporter activity	4	1.88E-05	0.01
GO:0005215	transporter activity	12	1.38E-05	0.01
GO:0034385	triglyceride-rich lipoprotein particle	4	1.74E-07	0.00
GO:0034361	VLDL particle	4	1.74E-07	0.00

VLDL = very low density lipoprotein. Gene# = the number of significant genes that are associated with this GO process.

To gain insights into pathways involved in the development of parasite resistance, we analyzed the differentially expressed genes using Ingenuity pathways analysis software, IPA. Among the 7 regulatory networks identified (data not shown), the primary function of 3 networks was related to lipid metabolism. The primary function of the 3^rd ^regulatory network was involved in antimicrobial response and inflammation. A total of 12 pathways were significantly impacted (*P *< 0.05) and possibly involved in the development of host resistance to parasitic infection in cattle (Table 5). FXR/RXR activation was the pathway most significantly impacted in resistant heifers (*P *= 3.66E-07) with at least 8 of the 203 differently expressed genes involved, including APOA1, APOB, APOC2, APOC3, FABP6, and IL18. The other pathways significantly impacted in resistant animals during parasitic infection included LXR/RXR activation (*P *= 2.78E-04), LPS/IL-1 mediated inhibition of RXR function (*P *= 8.80E-04), and arachidonic acid metabolism (*P *= 4.68E-03). In addition, acute phase response signaling was also impacted in resistant heifers (*P *< 0.05).

**Table 5:** Pathways significantly impacted during parasitic infection in resistant cattle.

Pathways	P value	Genes impacted
FXR/RXR Activation	3.66E-07	ABCC2, IL18, APOB, SCARB1, FABP6, APOC3, APOA1, APOC2
LXR/RXR Activation	2.78E-04	IL18, APOA4, APOA1, CD36, APOC2
LPS/IL-1 Mediated Inhibition of RXR Function	8.80E-04	ABCC2, SCARB1, FABP6, CYP3A4, APOC2, FMO5, SULT1B1
Arachidonic Acid Metabolism	4.68E-03	CYP4F2, AKR1C3, CYP3A4, CYP4B1, GGT1
Nicotinate and Nicotinamide Metabolism	7.08E-03	ENPP3, VNN1, NT5E, BST1
T Helper Cell Differentiation	1.56E-02	IL18, IL21R, CXCR5
Xenobiotic Metabolism Signaling	2.19E-02	ABCC2, CYP3A4, PPP2R2C, FMO5, MAPK11, SULT1B1
Inhibition of Angiogenesis by TSP1	2.45E-02	CD36, MAPK11
Interferon Signaling	2.74E-02	OAS1, MX1
Cell Cycle Regulation by BTG Family Proteins	2.89E-02	CCNE2, PPP2R2C
Pyrimidine Metabolism	3.05E-02	ENPP3, NT5E, ENTPD5, CTPS2
Acute Phase Response Signaling	4.21E-02	IL18, APOA1, RBP2, MAPK11

### Real-time RT- PCR confirmation

The expression of 10 genes at mRNA level in the fundic abomasum was examined using real-time RT-PCR. The mRNA level of cholecystokinin B receptor (CCKBR, NM_174262), a receptor for gastrin, was extremely low in the fundic abomasum. The mRNA levels of gastrin (GAST, NM_173915) and pepsinogen 5, group I (pepsinogen A) (PGA5, NM_001001600) were reliably detected. PGA5 expression level appeared to be higher in resistant animals. However, the difference was not statistically significant due to a large variation while no changes in gastrin mRNA level between susceptible and resistant animals were detected. The expression of MUC12 was barely detectable (≤ 40Ct). The mRNA levels of glucosaminyl (N-acetyl) transferase 3, mucin type (GCNT3, NM_205809), mucin 2 (MUC2, NM_001245997), and galectin 15-like (LGALS13, XM_593263) were moderately abundant but no differences were detected between susceptible and resistant animals, consistent with the RNAseq results.

**Figure 2:**
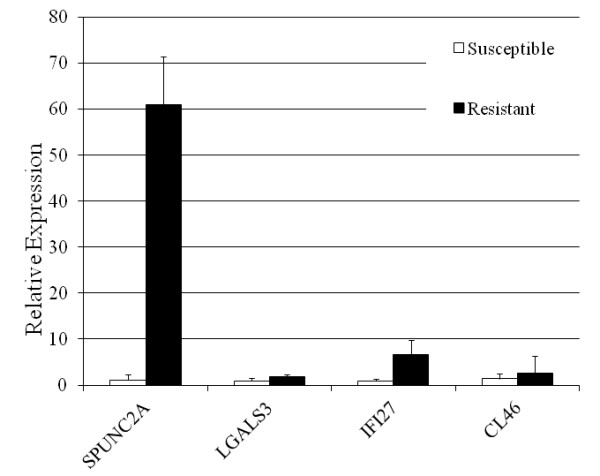
**The expression profiles of SPUNC2A, LGALS3, IFI27, and CL46 in the bovine fundic abomasum of susceptible and resistant heifers**. The expression value at the mRNA level was detected using quantitative RT-PCR. The expression value of one of the susceptible animals was set as 1.0. The fold change as calculated using the 2^-ΔΔCT ^method and normalized against the susceptible group (mean ± SD). SPUNC2A = BPI fold containing family A, member 2A (NM_174803); IFI27 = bovine putative ISG12(a) protein (NM_001038050); LGALS3 = lectin, galactoside-binding, soluble, 3 (NM_001102341), and CL46 = bovine collectin-46 (NM_001001856).

Expression levels of BPI fold containing family A, member 2A (BPIFA2A or SPUNC2A, NM_174803), bovine putative ISG12(a) protein (IFI27, NM_001038050), lectin, galactoside-binding, soluble, 3 (LGALS3, NM_001102341), and bovine collectin-46 (CL46, NM_001001856), were significantly higher in resistant animals, consistent with RNAseq data (Figure 2).

## Discussion

The parameters of resistance to GI nematode infections in cattle while yet to be precisely defined, generally include decreased worm establishment and reduced parasite fecundity. It has long been known that host genetic factors play a significant role in determining susceptibility and resistance. Among eight factors determining EPG variation, additive genetic variation is predominant and accounts for ~30% of the variation in EPG [24]. Estimates of heritability for parasite indicator traits in ruminants are phenotype-dependent. In small ruminants, the heritability of adult worm length at the end of the first grazing season is very strong at 0.62 [24], whereas the heritability of EPG is moderate, ranging from 0.14 to 0.33 in Creole goats [25]. In cattle, the heritability of EPG released during the 1^st ^grazing season is approximately 0.30 [26]. The ability of calves to recognize parasitic antigens is also under the control of host genetics [27]. Several studies suggest that there exist significant differences in the ability of cattle to resist GI nematode infections, and 3 major responder types can be readily identified in outbreed cattle populations [28,29]. Worm establishment (worm burden) is predominantly influenced by host responder types. The ability of intermediate and high responders to mount a more effective and rapid immune response compared to low responders is sustained after secondary infection, providing more evidence that genetics may play an important role in regulating host resistance. The finding that the different responder types, based on parasitological variables, also feature a different immune response is very interesting since this also provides the opportunity to study the influence of genetic components of the host immune response [28]. These observations have spurred efforts to develop resource populations and identify genes and QTL that underlie the resistance trait and to develop criteria for selective breeding [30].

A vigorous and effective mucosal immunity is essential for resistance to GI nematode infection in ruminants. The resistant phenotype is often manifested in the host transcriptome. For example, resistant sheep breeds are able to more rapidly up-regulate Th2 cytokines than susceptible breeds [31]. In Angus cattle, our evidence suggests that resistant heifers can better maintain inflammatory responses at the sites of infection, especially during early stages of infection [16]. In the current study, we conducted an in-depth transcriptomic analysis to identify molecular mechanisms that underlie the development of host resistance in cattle, taking advantage of a resource population developed via selective breeding. Our results suggest that among the 94 224 splice junctions identified, 133 were uniquely present in either resistant or susceptible cattle, possibly representing novel splicing variants that have implications in the development of host resistance. We identified 203 candidate genes that displayed significantly different numbers of sequences between resistant and susceptible animals at a combined cutoff value *P *< 0.05 and 2-fold.

The transcripts from 16 genes, including gastrin-releasing peptide (GRP) and macrophage-stimulating 1 (MST1), had a significantly higher number of sequences in susceptible cattle. GRP has been reported to be down-regulated by parasitic infection in a helminth-mouse system. Our results suggest that parasitic infection in susceptible cattle may have a negative impact on the host enteric nervous system that extends beyond its role in modulating normal functions of host epithelial, immune, and muscle cells [32].

Among the 187 genes with more abundant transcripts in the abomasum of resistant heifers, a notable feature was the up-regulation of various lectins. At least 4 lectins, such as bovine-specific collectin 46 (CL-46), C-type lectin domain family 12 member A (CLEC12A), galectin 3 (LGALS3), and intelectin 2 (ITLN2), had significantly more abundant transcripts in the abomasum of resistant cattle, which confirmed our previous study utilizing high-density DNA oligo arrays [33]. ITLN2 and several C-type lectins, such as collectin 11 (COLEC11), cattle-specific collectin-46, and conglutinin, as well as galectins were strongly up-regulated in the abomasal mucosa of immune cattle developed using multiple rounds of drug-attenuated infections [6,33]. ITLN2 expression is regulated by Th2 cytokine IL-4 [34]. Its elevated expression is observed in the sheep abomasums in response to *Teladorsagia circumcincta *infection, *Dictyocaulus filaria *natural infection [35], and *Haemonchus contortus *infection [36]. Most pertinent, this gene is naturally deleted in the genome of the susceptible mouse strain, C57BL/10, but is present in the genome of a nematode-resistant mouse strain, BALB/c, suggesting that this gene may serve a protective role in the innate immune response to *Trichinella *infection [37]. Cattle-specific collectin-46 has been suggested to provide the first line of defense against pathogens without eliciting a general inflammatory reaction [38]. Galectins also play an important role in innate immunity, including serving as receptors for pathogen-associated molecule patterns (PAMP), which is integral in recognizing carbohydrate moieties on the cell surface of parasites, activating various immune cells, participating in cytotoxicity, modulating innate immunity via binding to IgA, and promoting the reconstruction of damaged tissues as receptors for damage-associated molecular patterns (DAMP) [39]. Together, our results suggest that lectins may play an important role in invoking effective host immune responses and in the development of host resistance.

Our evidence also indicates that alterations in lipid metabolism may be necessary to the development of host resistance. The top function of 3 of the 7 regulatory networks identified was associated with lipid metabolism. GO terms associated with genes that were differently expressed between resistant and susceptible animals were also predominantly related to lipid metabolism (Table 4). Lipid metabolism is significantly regulated in the bovine small intestine. In response to *C. oncophora *infection, lipid balance in the GI tract during parasitic infection may be disrupted [17]. Polyunsaturated fatty acids (PUFA), especially those in omega-3 (n-3) and omega-6 (n-6) families, such as arachidonic acid and linoleic acid, have long been known to have strong immunomodulatory effects [40] and may serve as a potent inhibitor for Th1 response. In cattle, dietary supplementation with fish oil (omega-3 PUFA) results in a 24% reduction in EPG in calves that are infected with *O. ostertagi *and *C. oncophora *[41]. The treatment also leads to an increased percentage of immature parasites, indicating that PUFA may enhance protective immunity against parasitic infections. Interestingly, arachidonic acid metabolism was among the pathways most significantly impacted in resistant animals (Table 5). Arachidonic acid (AA) is one of the important PUFA-associated membrane phospholipids. When liberated from the plasma membrane, AA can be oxidized, via a series of enzymatic steps, to a variety of eicosanoids, including prostaglandins, thromboxanes, prostacycline, and leukotrienes. Eicosanoids act as signaling molecules and stimulate a variety of responses in their target cells, such as innate immune responses [42], inflammation, and smooth-muscle contraction. Dietary n-3 PUFA has been used to attenuate tissue AA levels and subsequent eicosanoid formation. Recently, worm killing activities of AA have been demonstrated [43]. In mice, a single oral dose of AA led to a significant reduction of total worm burden of *Schistosoma*. AA-mediated parasite killing is suggested to be due to excessive activation of parasite neutral sphingomyelinase, leading to sphingomyelin hydrolysis into ceramide and phosphorylcholine [43]. In addition, products of the 5-lipoxygenase pathway, a part of AA metabolism, are important mediators of inflammation. 5-lipoxygenase plays a major role in controlling parasite burden of *Trypanosoma cruzi *in mice [44]. Detailed link between lipid metabolism and the development of protective immunity and host resistance to parasitic infections in cattle is worthy of further investigation.

The three most significant pathways impacted in resistant animals are associated with retinoid X receptor (RXR). These pathways included FXR/RXR activation, LXR/RXR activation, and LPS/IL-1 mediated inhibition of RXR functions. RXR acts as a master coordinator of numerous signaling pathways [45] via dimerizing with other nuclear receptors, such as liver X receptor (LXR), farnesoid X receptor (FXR), and vitamin D receptor (VDR). This partnership exerts transcriptional control and leads to distinct functions ranging from cell proliferation and differentiation to lipid metabolism. In addition, RXR can bind to a variety of natural and synthetic ligands, including omega-3 unsaturated fatty acids [45], which in turn stimulate transcriptional activation by RXR partners. While retinoic acid receptors (RAR) bind all-*trans *retinoic acid (RA) and its 9-*cis *isomer (9-*cis *RA), which convey most of the activity of RA, only 9-*cis *RA and docosahexaenoic acid (DHA) are suggested to be endogenous RXR ligands [46]. RA can inhibit cytokine expression including reduction of TNFα, iNOS, IL-6, and IL-1β at the mRNA level [47]. Recently, it has been observed that LPS-specific regulatory networks in which NF-κB plays a critical role in the mouse mucosa overlap with the LPS/IL-1β mediated inhibition of RXR functions [48]. The importance of RXR in cattle during *C. oncophora *infection has also recently been recognized [17]. Our future work will focus on the mechanistic link between RXR-related signaling pathways and the development of host resistance to GI nematode infection in cattle.

## Competing interests

The authors declare that they have no competing interests.

## Authors' contributions

RWL conceived the study, conducted the experiment, analyzed the data, and drafted the manuscript. MR and AVC assisted in the experiment and contributed to the interpretation of results. All authors read and approved the final manuscript.

## Supplementary Material

Additional file 1 **Genes with significantly different read counts between resistant and susceptible cattle in response to parasitic infection**. A total of 203 genes met 2 criteria: unadjusted *P *value < 0.05 and 2-fold difference in normalized read counts between resistant and susceptible animals. Click here for file
